# Clinical characteristics and risk behavior as a function of HIV status among heroin users enrolled in methadone treatment in northern Taiwan

**DOI:** 10.1186/1747-597X-6-6

**Published:** 2011-04-08

**Authors:** Tony Szu-Hsien Lee, Hsi-Che Shen, Wei-Hsin Wu, Chun-Wei Huang, Muh-Yong Yen, Bo-En Wang, Peing Chuang, Chien-Yu Shih, Ying-Chun Chou, Yi-Lien Liu

**Affiliations:** 1 Department of Health Promotion and Health Education, National Taiwan Normal University, No. 162, He-ping East Road, Section 1, Taipei 10610, Taiwan; 2 New Taipei City Hospital and Taiwan Medical University No.198, Yingshi Rd., Banqiao City, New Taipei City 241, Taiwan; 3 Department of Psychiatry, Keelung Hospital, Department of Health, No 268 Sin 2nd Rd, Keelung City 20148 Taiwan; 4 Department of Psychiatry, Lotung Poh-Ai Hospital, 83 Nan Chang St., Lotung, Yilan 265, Taiwan; 5 Department of Infection, Taipei City Hospital, Branch for Disease Control and Prevention, No 100 Quan-Ming Road Taipei 10844 Taiwan; 6 Keelung City Health Bureau, No.266, Sin 2nd Rd., Sinyi District, Keelung City 201, Taiwan; 7 Public Health Bureau, Taoyuan County, No.1,Shianfu Rd, Taoyuan City, Taoyuan County 33001 Taiwan

## Abstract

**Background:**

Methadone treatment was introduced in Taiwan in 2006 as a harm-reduction program in response to the human immunodeficiency virus (HIV), which is endemic among Taiwanese heroin users. The present study was aimed at examining the clinical and behavioral characteristics of methadone patients in northern Taiwan according to their HIV status.

**Methods:**

The study was conducted at four methadone clinics. Participants were patients who had undergone methadone treatment at the clinics and who voluntarily signed a consent form. Between August and November 2008, each participant completed a face-to-face interview that included questions on demographics, risk behavior, quality of life, and psychiatric symptoms. Data on HIV and hepatitis C virus (HCV) infections, methadone dosage, and morphine in the urine were retrieved from patient files on the clinical premises, with permission of the participants.

**Results:**

Of 576 participants, 71 were HIV positive, and 514 had hepatitis C. There were significant differences between the HIV-positive and HIV-negative groups on source of treatment payment, HCV infection, urine test results, methadone dosage, and treatment duration. The results indicate that HIV-negative heroin users were more likely to have sexual intercourse and not use condoms during the 6 months prior to the study. A substantial percent of the sample reported anxiety (21.0%), depression (27.2%), memory loss (32.7%), attempted suicide (32.7%), and administration of psychiatric medications (16.1%). There were no significant differences between the HIV-positive and HIV-negative patients on psychiatric symptoms or quality of life.

**Conclusions:**

HIV-positive IDUs were comorbid with HCV, indicating the need to refer both HIV- and HCV-infected individuals for treatment in methadone clinics. Currently, there is a gap between psychiatric/psychosocial services and patient symptoms, and more integrated medical services should be provided to heroin-using populations.

## Background

Many countries in the world are grappling with problems of drug use, and Taiwan is no exception. In the past decade, there has been a resurgence of opiate (heroin) use in Taiwan, and this increase poses great challenges for health professionals and policy makers. According to the Taiwan Center for Disease Control (CDC), the number of people using heroin has increased during the past 5 years, and the incidence of contracting the human immunodeficiency virus (HIV) and/or the hepatitis C virus (HCV) has increased tenfold [1]. At the end of 2009, the proportion of HIV cases related to Taiwanese injection drug users (IDUs) was 34% [2], and the percentage of IDUs that had contracted HCV was about 80-90% [3]. Because oral ingestion of methadone can help heroin users prevent HCV and HIV infection and reduce the negative consequences of heroin use by managing withdrawal syndromes and cravings [4-6], methadone maintenance treatment was adopted in Taiwan, and it expanded rapidly as part of a harm-reduction policy aimed at stemming the spread of HIV [7]. According to the Taiwan CDC (2010), there are currently 100 hospital clinics providing methadone treatment, supplemented by 22 satellite community health centers. Together, these units serve more than 11,000 patients.

Because in Taiwan the primary goal of methadone maintenance treatment is to stem the spread of HIV rather treat heroin dependence, the use or possession of heroin is still illegal. Offenders can choose either to take methadone for at least one year as an outpatient in a hospital or be incarcerated in a residential detoxification or rehabilitation center administered by the Taiwan Ministry of Justice. Although methadone maintenance is widely acknowledged as an effective treatment for heroin dependence and the reduction of HIV infections in Western societies [5], only recently has it become established in Taiwan, where its utility has been novel to psychiatrists, methadone patients and stakeholders. Thus, it is important to determine who participates in methadone treatment in Taiwan and if there are differences in the HIV status of heroin users. The present study was conducted to examine HIV serostatus in relation to the demographics, clinical characteristics, risk behavior, and quality of life of methadone patients in northern Taiwan.

## Methods

### Participants and procedure

The participants were recruited through four methadone clinics in New Taipei City, Taipei city, Keelung and I-Lan county, all in northern Taiwan. The inclusion criteria for participants were as follows: at least 18 years old, literate, using heroin in the past 6 months, and currently enrolled in a methadone treatment program. The research was approved by the Institutional Review Board of Human Subjects Protection at Taipei Medical University (approval number: P960205) and Taipei City Hospital (approval number: TCHIRB-970404-E). After explaining the purpose of the research to the methadone patients, the participants voluntarily signed a consent form and took a face-to-face interview, which was conducted between August and November 2008. Interviews were conducted in a private room provided by the clinic and took approximately 15 minutes to complete. At the time of data collection, participants had already been engaged in methadone treatment. All participants received remuneration of 100 Taiwan dollars (approximately 3 US dollars) for their participation.

### Measures

A semi-structured questionnaire was used to obtain information about demographics, quality of life, HIV risk behavior, and psychiatric symptoms. Their treatment right, anonymity and confidentiality were assured to participants so that questions can be responded comfortably and truthfully.

#### Demographics and clinical characteristics

The demographic items asked about the participant's age, age at first heroin use, gender, education, source of methadone payment, and employment. Before admission to methadone treatment, the patients were asked by their physician to take tests for HIV, HCV, and morphine if they had not previously tested positive for the factor. The psychiatrists' determination of the initial daily dose of methadone and the payment was based on the patients' drug-using history and the results of the tests mentioned above. For patients confirmed as HIV positive, the cost of the methadone treatment was fully covered by the Taiwan government. All patients were reassessed monthly so their doctor could adjust the daily dose. After obtaining patient consent, records for the variables examined in the study were retrieved from patient files on the hospital's computer systems. The data included HIV status, HCV status, morphine from urine tests, and methadone dosage at intake. Data from the urine tests and methadone dosage were also collected at the times of the interview.

#### Quality of life

We used the World Health Organization Quality of Life Assessment-Brief Version (WHOQOL-BREF), which was developed to measure overall quality of life and general health status [8,9]. The WHOQOL-BREF measures four health domains (physical, psychological, social and environmental). The instrument has shown excellent internal consistency (Cronbach's alpha = 0.97), as well as good test-retest reliability over 2 weeks for the four domains (0.68 to 0.85) [9]. In the current study, Cronbach's alpha was 0.91. The four-domain structure was validated by exploratory and confirmatory factor analysis, with factor loadings > 0.40 for the retained items and CFI > 0.90 [9]. These latter results demonstrate adequate validity.

#### Risk behavior and psychiatric symptoms

Respondents indicated their risk behavior 6 months period before the interview. Sex-related and drug-related risk behavior were assessed by five items: 1) having sexual coitus during the last 6 months, 2) using a condom during last sexual intercourse, 3) frequency of condom use during the last 6 months, 4) heroin use during last 6 months, and 5) needle sharing during last 6 months. Psychiatric symptoms were measured in the past 30 days before the interview by five questions covering 1) anxiety, 2) depression, 3) memory loss, 4) attempted suicide, and 5) psychiatric medications prescribed.

### Statistical analysis

Statistical analyses were conducted using SPSS version 16.0 [10]. Differences between the two HIV groups in the categorical proportions for demographic factors, clinical characteristics, types of risk behavior, and psychiatric symptoms were evaluated using chi-square tests, or Fisher exact tests if the sample was small. Comparisons of the two HIV groups on continuous variables, including age, age at first heroin use, methadone dose, treatment duration and quality of life were performed using independent sample t tests. Items with missing values were omitted from the analysis. The criterion for statistical significance was set at p < 0.05, two-tailed.

## Results

### Demographics and clinical characteristics

Of the 599 patients recruited for the study, 576 completed the consent forms and interviews. Of the latter, 71 (12%) were HIV positive. Table 1 presents participant demographics and clinical characteristics according to HIV status. The mean age of the participants was 40.6 years, and the mean age at first heroin use was 27.2 years. Treatment fee was fully covered by government for the HIV-positive patients in contrast to 35.4% for the HIV-negative group. In addition, 71% had completed 9 years of compulsory education, and 66% were employed at the time of assessment. No other significant differences were found between the demographics of the HIV-positive and HIV-negative participants.

**Table 1:** Demographic and clinical characteristics as a function of the HIV status of methadone patients from four hospitals (N = 576).

		HIV-positive	HI- negative	Total
		
		n = 71 (12%)	n = 505 (88%)	
Age	(years, mean ± SD)	39.3 ± 7.8	40.8 ± 9.4	40.6 ± 9.3
Age at first heroin use	(years, mean ± SD)	25.9 ± 6.2	27.4 ± 7.5	27.2 ± 7.4
Gender	Male Female	67 (90%) 7 (10%)	438 (87%) 64 (13%)	503 (87%) 73 (13%)
Source of treatment fee	Self-paid	0 (0%)	330 (65.3%)	330 (57.3%)**
	Government sponsor	71 (100%)	175 (35.4%)	246 (42.7%)
Education	Less than 9 years At least 9 years	22 (31%) 49 (69%)	142 (28%) 363 (72%)	164 (29%) 412 (71%)
Employed (n = 549)^a^	No	30 (44%)	157 (33%)	187 (34%)
	Yes	38 (56%)	324 (67%)	362 (66%)
HCV (n = 552) ^a^	Negative	0 (0%)	38 (8%)	38 (7%)**
	Positive	66 (100%)	442 (92%)	514 (93%)
Morphine at intake (n = 545) ^a^	Negative	31 (45%)	145 (31%)	176 (32%)*
	Positive	38 (55%)	331 (69%)	369 (68%)
Morphine at interview (n = 506) ^a^	Negative	48 (76%)	294 (66%)	342 (68%)
	Positive	15 (24%)	149 (34%)	164 (32%)
Average methadone dose at intake	(mg, mean ± SD)	37.5 ± 20.4	38.7 ± 20.3	38.5 ± 20.3
Average methadone dose at interview	(mg, mean ± SD)	60.4 ± 35.0	48.2 ± 30.9	49.8 ± 31.7**
Time from intake to interview	(days, mean ± SD)	218 ± 164	179 ± 146	184 ± 149*

* p < 0.05.

** p < 0.01.

^a ^Missing values were omitted from the statistical analyses.

Note: χ^2 ^and t tests were used to examine differences between the HIV groups on categorical and continuous variables respectively. A Fisher exact test was performed to examine the relationship between HCV and HIV.

The two HIV groups differed significantly on HCV and morphine at intake and on methadone dosage at interview (Table 1). Interestingly, 514 (93%) were HCV positive, and every HIV-positive patient tested positive for HCV. In addition, 92% of the HIV-negative participants were positive for HCV. The between-HIV-group difference was significant for HCV prevalence by Fisher's exact test (p < 0.01). The urine tests showed that more than 70% of the patients in the HIV-negative group tested positive for morphine at intake, compared to 55% in the HIV-positive group, χ^2^(1, N = 544) = 6.08, p < 0.05. The average dose of methadone at intake was 38.5 ± 20.3 mg and the dosage increased to 49.8 ± 31.7 mg by the time of the interview. At that time, the mean dose of methadone for the HIV-positive group was significantly higher than for the HIV-negative group, t(543) = 2.95, p < 0.01. Also, the HIV-positive group spent on average significantly more days in methadone treatment than the HIV-negative group (218 days vs. 179 days); t(553) = 2.05, p < 0.05.

### HIV risk behavior, psychiatric symptoms, and quality of life

Table 2 shows the differences between the HIV-positive and HIV-negative groups on risk behavior, psychiatric symptoms, and quality of life. The groups differed significantly on sexual intercourse within the last 6 months, χ2(1, N = 528) = 19.7, *p *<.01, condom use at last sexual encounter, χ2(1, N = 460) = 15.301, p < 0.01, frequency of condom use within the last 6 months, χ2(2, N = 390) = 13.164, p < 0.01, and heroin injection within the last 6 months, χ2(1, N = 518) = 4.309, p < 0.05. Specifically, 90% of the HIV-negative patients who were sexually active had sexual intercourse within the last 6 months compared to 70% of the corresponding HIV-positive patients. Likewise, 54% of the sexually-active HIV-negative patients did not use condoms during their last sexual intercourse, compared with 23% of the corresponding HIV-positive patients. Whereas 42% of the sexually active HIV-positive patients reported that they always or frequently used condoms during the last 6 months, 24% reported that they used condoms only sometimes during this period, and 34% reported that they never used them during this period (Table 2). In contrast, 18%, 22%, and 60% of HIV-negative patients reported that they always/frequently, sometimes, and never used condoms during the last 6 months, respectively.

**Table 2:** HIV risk behavior, psychiatric symptoms, and quality of life as a function of HIV status.

		HIV-positive	HIV-negative	Total
Sexual Risk				
Engaging in sex within last 6 months (n = 528) ^a^	Yes	47 (70%)	413 (90%)	460 (87%) **
	No	20 (30%)	48 (10%)	68 (13%)
Condom use at last sexual encounter (n = 460)	Yes	36 (77%)	192 (46%)	228 (50%) **
	No	11 (23%)	221 (54%)	232 (50%)
Frequency of condom use within last 6 months (n = 391) ^a^	Always/Frequently	16 (42%)	66 (18%)	82 (21%) **
	Often/Sometimes	9 (24%)	76 (22%)	85 (22%)
	Few/Never	13 (34%)	211 (60%)	224 (57%)
Drug-related risk				
Heroin injection within last 6 months (n = 518) ^a^	Yes	54 (81%)	403 (89%)	457 (88%) *
	No	13 (19%)	48 (11%)	61 (12%)
Sharing syringes during last 6 months (n = 457) ^a^	Always/Frequently	2 (4%)	3 (1%)	5 (1%)
	Often/Sometimes	5 (9%)	19 (5%)	24 (5%)
	Few/Never	47 (87%)	381 (94%)	428 (94%)
Psychiatric Symptoms (n = 523) ^a^				
Anxiety	No	51 (78.5%)	362 (79.0%)	413 (79.0%)
	Yes	14 (21.5%)	96 (21.0%)	110 (21.0%)
Depression	No	47 (72.3%)	334 (72.9%)	381 (72.8%)
	Yes	18 (27.7%)	124 (27.1%)	142 (27.2%)
Memory loss	No	46 (70.8%)	306 (66.8%)	352 (67.3%)
	Yes	19 (29.2%)	152 (33.2%)	171 (32.7%)
Attempted suicide	No	46 (70.8%)	306 (66.8%)	352 (67.3%)
	Yes	19 (29.2%)	152 (33.2%)	171 (32.7%)
Psychiatric medication prescribed	No	52 (80.0%)	386 (84.5%)	438 (83.9%)
	Yes	13 (20.0%)	71 (15.5%)	84 (16.1%)
Quality of life (n = 551) ^a^	Range 0 to 100	(mean ± SD)	(mean ± SD)	(mean ± SD)
Physical domain		58.22 ± 17.57	56.80 ± 16.07	56.98 ± 16.25
Psychological domain		45.27 ± 15.91	48.91 ± 14.77	48.46 ± 14.95
Social domain		50.51 ± 19.05	54.02 ± 18.54	53.58 ± 18.62
Environment domain		53.42 ± 16.79	51.45 ± 17.13	51.69 ± 17.08

* p < 0.05.

** p < 0.01.

^a ^Missing values were omitted from the statistical analyses.

Note: χ^2 ^and t tests were used to examine differences between the HIV groups on categorical and continuous variables respectively. A Fisher exact test was performed to examine the relationship between syringe sharing and HIV.

The results show that 88% of the participants reported that they had injected heroin within the last 6 months. This percentage was higher in the HIV-negative group than the HIV-positive group, χ2(1, N = 518) = 4.309, p < 0.05. In addition, 94% of the patients reported that they rarely or never shared their syringes with others during the last 6 months. However, 7 HIV-positive participants reported sharing syringes during this period.

Regarding psychiatric symptoms 30 days prior to the interview, 21% of the participants experienced anxiety, 27.2% experienced depression, 32.7% had difficulty with memory, 32.7% attempted suicide, and 16.1% took psychiatric medications within the past 30 days. No significant differences were found between psychiatric symptoms and HIV status.

As for quality of life, of the four domains measured by the WHOQOL-BREF, the methadone patients scored lowest on psychological (mean = 48.46) and highest on physical (mean = 56.98). However, no significant differences in quality of life were found between the HIV-positive and HIV-negative groups on the overall scale or any of the four domains.

## Discussion

This study was aimed at determining the demographics and clinical characteristics of methadone patients in northern Taiwan as a function of their HIV status. We found no demographic differences between the HIV-positive and HIV-negative groups. The demographics of the patients in the present study were similar to those in other studies of heroin use [11], except that our respondents were about 5 to 7 years older than those in the other studies when they began using heroin [5,12]. But, the mean age of first heroin use in this study (27.2 ± 7.4 years) was similar to a previous study (25.4 ± 6.7 years) conducted among 155 treatment-seeking heroin abusers in Taiwan [13].

With respect to clinical characteristics, there were significant differences between our HIV-positive and HIV-negative groups on HCV infection, morphine in the urine and methadone dosage at the time of the interview. Because IDUs commonly share injection equipment (needles), and HCV and HIV are transmitted mainly through blood and serum, IDUs are highly susceptible to HCV and HIV [4,5,14,15]. Global estimates of the prevalence of HCV infection in IDUs have been highest for Asia [16]. In most Asian countries, including Taiwan, drug users are tested for HCV but receive no treatment for it [17]. As they do for HIV testing and counseling of men who have sex with other men, hospitals should design and implement programs for the behavior counseling and education programs about HCV and health generally.

In our study, we found that psychiatrists increased the average daily dose of methadone to 60 mg in HIV-positive patients but not in HIV-negative patients. Also, the HIV-positive patients started using heroin earlier and stayed longer in treatment. There are two plausible explanations. One is that the difference in dosing is because the severity of dependence between these two groups may not be the same at the intake. Another explanation may reflect the fact that since the harm-reduction policy was initiated by the Taiwan government in 2006, psychiatrists in Taiwan have been professionally trained and educated in a way that treats methadone as an HIV prevention strategy rather than as a treatment for substance abuse. Policy makers may want to reconceptualize methadone as a relatively effective treatment for opioid dependence, given that the number of reports of new HIV cases related to heroin use has been decreasing in Taiwan [2]. A previous study found that methadone treatment can result in longer treatment stays and an opioid abstinence rate twice as high as with psychosocially enriched detoxification [18]. Further evidence of the effectiveness of methadone treatment comes from our finding that prescribing 60 mg or more of methadone can result in a longer stay in treatment than prescribing a lower dose. This finding is consistent with a previous study of 428 methadone patients in which it was found that a daily methadone dose of 60 mg/day or above was associated with longer retention in treatment [19]. Hence, both policy makers and psychiatrists should redefine methadone treatment as a medication-assisted therapy that is aimed at, but not limited to, the reduction of HIV infection among heroin users.

Another policy issue is how much heroin users should pay for their methadone treatment if the policy goal is to reduce the harm caused by drug use. According to Taiwan's HIV Prevention Act, anti-retroviral therapy (commonly known as cocktail therapy) and methadone treatment for HIV-positive patients are fully reimbursed by the Taiwan Department of Health. Combined with our finding that the average duration of treatment was longer for the HIV-positive group than for the HIV-negative group, this finding indirectly supports the hypothesis that people stay longer in methadone treatment if it is free. A previous study of 557 injection drug users likewise found that free treatment can result in a greater likelihood of patients' remaining in treatment for a minimum of 90 days; longer continuation of treatment was also associated with better patient outcomes [20]. Policy makers in Taiwan may want to explore further whether free treatment can result in better outcomes in terms of reducing the bio-psycho-social harm of heroin use.

Our results for risk behavior show that the HIV-negative participants were more likely to be sexually active and less likely to use condoms during sexual intercourse than the HIV-positive participants. These results are consistent with some Western studies [21,22] suggesting that most HIV-infected drug users avoid or abstain from risky behavior that may result in the transmission of HIV. There have been mixed results, however, concerning whether HIV-infected individuals actually engage in safer behavior. Some studies found that HIV-positive drug users continued their risky practices. For example, a study on a small sample of 50 HIV-positive IDUs found that 66% continued to engage in either sex-related or drug-related risky sexual behavior even after they were aware of being HIV-positive, and 42% admitted to having engaged in both types of risk behavior [23]. In another study of 3723 HIV-infected persons, about 79% of the 304 participants who reported injecting drugs in the past year were sexually active, 50% had multiple partners, and 52% engaged in unprotected intercourse during the past 3 months. We also found that 21 out of our 71 HIV-infected respondents were continuing to have sexual coitus without always using condoms. Thus, it is imperative for substance use programs to begin setting treatment goals and adopting treatment strategies that encourage HIV-infected individuals to always adopt safer sexual practices. These interventions must also be sensitive to the special needs and circumstances of the drug-using population. HIV risk-reduction and behavioral counseling have not yet been incorporated in methadone treatment programs in Taiwan. If personnel and funding are limited, we recommend that such counseling restrict its focus to HIV-positive heroin users at methadone clinics.

Providing methadone to heroin users not only limits the needle sharing that can stem the spread of infectious diseases, but it also treats addiction and the drug-related effects of heroin dependence, which affects the drug user's quality of life. The mean quality-of-life scores in our study ranged from 48.46 to 56.98 on a 0 to 100 scale. Our respondents scored 2-6 points lower than people with hepatitis and 13 points lower than the general population of Taiwan [25]. The lower quality-of-life mean for heroin users may partially be the effect of labeling, as well as the strong social stigma attached to drug addiction [26]. Drug users also are likely to have poor social/family support, which can negatively affect their mental health and social skills, thereby further lowering their quality of life [27]. In our study, we found no differences in quality of life between the HIV-positive and HIV-negative patients. However, there may be real differences that were obscured by the labeling and stigma mentioned above. In any case, the medical community should consider eliminating discrimination toward drug users and have more empathy for Taiwanese who suffer from the effects of heroin use.

Examination of the psychiatric symptom data reveals that one fourth to one third of the respondents receiving methadone treatment needed additional medical services for their psychiatric problems. Consistent with other studies [28], the rates of depression and depressive symptoms were similar for our HIV-positive and HIV-negative groups. Our results indicate a high prevalence of psychiatric comorbidity among Taiwan heroin users, and a substantial number of our respondents had received no treatment for their anxiety, depression, or suicide attempts. These symptoms might be attributable to psychosocial or behavioral factors such as poor social support, a low sense of personal control, low socioeconomic status, and low self-esteem [28]. Our results highlight the importance of combining methadone treatment with psychosocial services, which are currently not included in substance abuse treatment. Examples of the services needed to alleviate depression and anxiety are the strengthening of family support, the elicitation of emotional catharsis, and cognitive psychotherapy.

The present study has a few limitations. As our participants were recruited from exclusively four methadone clinics in northern Taiwan, they may not constitute a representative sample of Taiwanese heroin users. Even though our clinic-based sample was large, our participants may differ in some relevant respects from the general population of heroin users. Second, because risk behavior was assessed through a questionnaire, it was not possible to validate whether the patients answered the questions truthfully. We attempted to reduce this problem by providing our participants with confidentiality.

## Conclusions

Despite the limitations of our study, several findings provide valuable information on how to improve methadone treatment and service delivery in Taiwan and perhaps elsewhere. The HIV-positive patients in our sample, all of whom received free treatment, were prescribed higher doses of methadone and stayed longer in treatment than the HIV-negative patients. Second, HCV infection was prevalent among the heroin users, and all the HIV-positive participants had contracted HCV. Third, the HIV-negative patients were more likely to be sexually active and less likely to use condoms during sexual intercourse than the HIV-positive patients. However, 21(29.6%) and 7(10%) of the HIV-positive patients continued to engage in sex- and drug-related risk behavior even after being informed of their HIV status. Fourth, there was a high prevalence of psychiatric comorbidity among the heroin users, which highlights the need to pay more clinical attention to the integration of methadone treatment and psychiatric medication. Changing psychiatrists' perception of methadone therapy from harm reduction to a treatment for heroin dependence, and integrating psychosocial services and HCV treatment in clinical practice, can improve treatment outcomes related to the harmful effects of drug use. Finally, more research is needed on the effect of making methadone treatment free.

## Competing interests

The authors declare that they have no competing interests.

## Authors' contributions

All the authors contributed to the planning, execution, and/or reporting of this study. TSHL conceived the study and drafted the manuscript. HCS, WHW, CWH, and MYY participated in the study design, and they coordinated and drafted the introduction and discussion sections of the manuscript. BEW, PC, CYS, YCC, and YLL performed the data analyses, drafted the results and discussions sections, and prepared the tables. All the authors read and approved the final manuscript.
